# 11β-Hydroxysteroid Dehydrogenase Activity in the Brain Does Not Contribute to Systemic Interconversion of Cortisol and Cortisone in Healthy Men

**DOI:** 10.1210/jc.2014-3277

**Published:** 2014-11-13

**Authors:** Alixe H.M. Kilgour, Scott Semple, Ian Marshall, Peter Andrews, Ruth Andrew, Brian R. Walker

**Affiliations:** MRC Centre for Cognitive Aging and Cognitive Epidemiology (A.H.M.K.), Geriatric Medicine Unit, and Centre for Clinical Brain Sciences (S.S., I.M., P.A.), University of Edinburgh, Edinburgh, United Kingdom; Clinical Research Imaging Centre (S.S.) and BHF Centre for Cardiovascular Science (S.S., R.A., B.R.W.), Queen's Medical Research Institute, University of Edinburgh, Edinburgh, United Kingdom; Critical Care (P.A.), Western General Hospital, NHS Lothian University Hospitals Division, Edinburgh EH4 2XU, United Kingdom

## Abstract

**Context and Objective:**

11β-hydroxysteroid dehydrogenase type 1 (11βHSD1) catalyses regeneration of cortisol in liver, adipose tissue, and skeletal muscle, making a substantial contribution to circulating cortisol as demonstrated in humans by combining stable isotope tracer infusion with arteriovenous sampling. In the brain, 11βHSD1 is a potential therapeutic target implicated in age-associated cognitive dysfunction. We aimed to quantify brain 11βHSD1 activity, both to assess its contribution to systemic cortisol/cortisone turnover and to develop a tool for measuring 11βHSD1 in dementia and following administration of 11βHSD1 inhibitors.

**Design, Setting, and Participants:**

With ethical approval and informed consent, 8 healthy men aged 38.1 years (sd 16.5) underwent an ECG-gated phase-contrast magnetic resonance scan to quantify internal jugular vein blood flow and were infused with 1,2 [^2^H]_2_-cortisone and 9,11,12,12 [^2^H]_4_-cortisol for 3 h before samples were obtained from the internal jugular vein and an arterialized hand vein. Steroids were quantified by liquid chromatography-tandem mass spectrometry.

**Main Outcome Measures and Results:**

Steady state tracer enrichments were achieved and systemic indices of cortisol/cortisone interconversion were consistent with previous studies in healthy men. However, there was no measurable release or production of cortisol, 9,12,12 [^2^H]_3_-cortisol or cortisone into the internal jugular vein.

**Conclusions:**

Although cerebral 11βHSD1 reductase activity may be greater in cognitively impaired patients, in healthy men any contribution of 11βHSD1 in the brain to systemic cortisol/cortisone turnover is negligible. The influence of 11βHSD1 in the brain is likely confined to subregions, notably the hippocampus. Alternative approaches are required to quantify pharmacodynamics effects of 11βHSD1 inhibitors in the human brain.

The 11β-hydroxysteroid dehydrogenases (11βHSDs) are intracellular enzymes that catalyze interconversion of inactive cortisone and active cortisol; 11βHSD type 1 is a predominant reductase, regenerating cortisol from cortisone, while 11βHSD type 2 is an exclusive dehydrogenase, inactivating cortisol to cortisone. An extensive literature documents the role of 11βHSDs in regulating intracellular glucocorticoid concentrations and hence modulating tissue-specific activation of corticosteroid receptors (reviewed in 1, 2). 11βHSD1 amplifies glucocorticoid action in the liver, adipose tissue, inflammatory cells, and vasculature, providing a therapeutic target for inhibition in type 2 diabetes. 11βHSD2 limits cortisol action, and thereby facilitates aldosterone action in the distal nephron and a few other sites, explaining the mineralocorticoid excess state which ensues when 11βHSD2 is inhibited by licorice.

Both 11βHSD isozymes also contribute to systemic turnover of cortisol. Activity of the two isozymes has been quantified in humans using stable isotope (deuterated) glucocorticoid tracers (Figure 1). Production of cortisone can be measured by the rate of dilution of 1,2-[^2^H]_2_-cortisone (d2-cortisone) by cortisone, and is inhibited by licorice (3). A more complex tracer is used to measure regeneration of cortisol by 11βHSD1: 9,11,12,12-[^2^H]_4_-cortisol (d4-cortisol) is infused and production of cortisol is measured by the rate of dilution of d4-cortisol by cortisol (an index of net cortisol production from all sources, including the adrenal gland) and by 9,12,12-[^2^H]_3_-cortisol (d3-cortisol, a specific measure of cortisol regeneration by 11βHSD1) (4). Using these tracers in combination with selective venous catheterization and measurement of blood flow has allowed quantification in humans of cortisol-cortisone interconversion in splanchnic (3, 5, 6), subcutaneous adipose (3, 7), and skeletal muscle (3) circulations, and exclusion of significant 11βHSD activity in the myocardium (8). These studies reveal rapid shuttling between cortisol and cortisone such that in healthy men at rest, remarkably, the magnitude of extra-adrenal regeneration of cortisol by 11βHSD1 is greater than the magnitude of adrenal cortisol secretion. They also suggest, surprisingly, that 11βHSD1 may catalyze both reductase and dehydrogenase activity in vivo, resulting in “recycling” between cortisol and cortisone (detected by dilution of d2-cortisone by cortisone and by simultaneous dilution of d4-cortisol by d3-cortisol) even in tissues where 11βHSD2 is not expressed (3).

**Figure 1.**
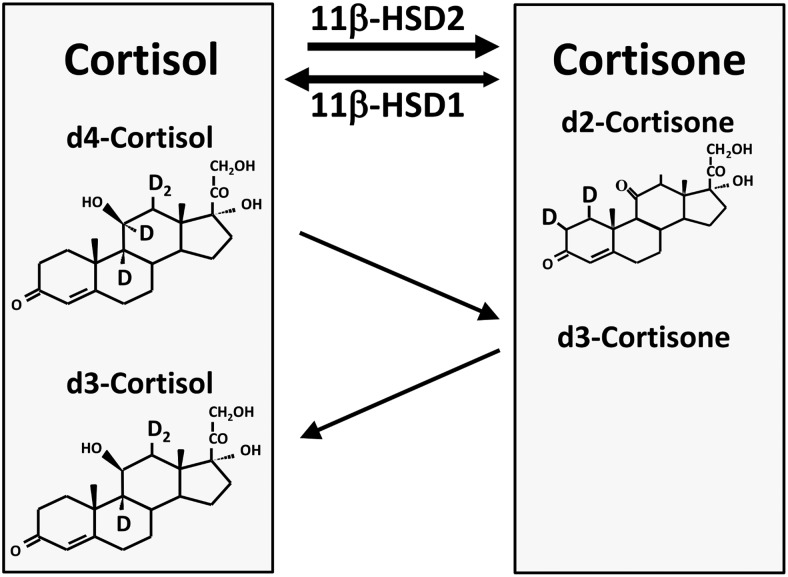
Stable isotope tracers for measuring cortisol-cortisone interconversion by 11β-HSDs. 11β-HSD2 is a unidirectional enzyme catalyzing the dehydrogenase conversion of cortisol to cortisone. 11β-HSD1 is a potentially reversible enzyme catalyzing interconversion of cortisol and cortisone, predominantly in the reductase (cortisone to cortisol) direction. The shaded boxes on left and right represent the circulating pools of cortisol and cortisone, respectively. Production of cortisone can be measured by infusing a tracer, d2-cortisone, into the cortisone pool and measuring its dilution by cortisone. Similarly, production of cortisol can be measured by infusing a tracer, d4-cortisol, into the cortisol pool. When d4-cortisol is metabolized by 11β-HSD2, the deuterium in the 11α position is removed, producing d3-cortisone; when this d3-cortisone is converted back to cortisol by 11β-HSD1 it is highly unlikely that a deuterium rather than a proton will be reincorporated, so that d3-cortisol is produced. Dilution of d4-cortisol with d3-cortisol therefore indicates 11β-HSD1 reductase activity.

11βHSDs may also play key roles in the brain (9). 11βHSD2 is expressed in the developing, but not the adult brain (10). 11βHSD1 is expressed more widely in the adult brain, and notably in the prefrontal cortex, hippocampus, and cerebellum, a distribution confirmed in humans (11). Increased local regeneration of cortisol by 11βHSD1 may cause glucocorticoid-dependent neurotoxicity and hence contribute to cognitive aging and dementia, while inhibition of 11βHSD1 has been proposed as a therapeutic strategy to treat age- and dementia-associated cognitive dysfunction. In mice, 11βHSD1 levels in the hippocampus and parietal cortex rise with age and correlate with impaired cognitive performance, while transgenic overexpression of 11βHSD1 in the forebrain accelerates age-associated cognitive decline (12). Conversely, 11βHSD1 knockout mice are protected from age-related learning impairment (13, 14). Moreover, selective 11βHSD1 inhibitors, after either systemic or intracerebroventricular administration, improve cognitive function in aged mice (14). Indeed in humans, the nonselective 11βHSD inhibitor carbenoxolone improved cognitive performance in healthy elderly and diabetic men (11). However, a recent phase II clinical trial of a selective 11βHSD1 inhibitor, ABT384, in patients with mild-to-moderate Alzheimer's disease was halted prematurely because of lack of efficacy (15). It is uncertain whether this reflected selection of patients whose cognitive dysfunction is no longer responsive to reducing cortisol action, or inadequate inhibition of brain 11βHSD1 by ABT384.

Against this background, quantification of brain 11βHSD1 activity in vivo in humans would be highly desirable. Here, we aimed: (i) to determine whether, as a major organ by mass, brain 11βHSD1 contributes to cortisol/cortisone turnover in vivo; (ii) to establish whether 11βHSD1, as the only 11βHSD isozyme expressed in an adult brain (11), catalyzes only regeneration of cortisol or also recycling between cortisol and cortisone; (iii) to provide a tool with which to quantify changes in human brain 11βHSD1 activity with dementia, and to use as a pharmacodynamics biomarker to quantify enzyme inhibition during clinical development of selective 11βHSD1 inhibitors for treating dementia. We therefore extended our previous studies using arteriovenous sampling with stable isotope tracer infusions in vivo to quantify 11βHSD activities in the human brain.

## Materials and Methods

### Participants

Participants were recruited through advertisements in the local press and around the University campus. Participants were healthy male volunteers between 18 and 70 years old. Exclusion criteria were glucocorticoid medication (by any route of administration) within the past 3 months; diabetes mellitus, cerebrovascular disease or other significant chronic illness; history of recent heavy alcohol or illegal drug use; current use of any immunosuppressive medication; abnormal screening liver, thyroid, renal or coagulation function (ie, INR > 1.5 or platelets < 50 × 10^9^/L) or abnormal full blood count or random blood glucose; research participant in the previous 3 months; any contraindication to magnetic resonance (MR) imaging. The study complied with the Declaration of Helsinki; ethical approval was obtained from the local Research Ethics Committee (Scotland A REC, reference 12/SS/0079) and all participants gave written informed consent.

### Reagents

Reagents were obtained from Sigma, Steraloids, or VWR. 1,2 [^2^H]_2_-cortisone (d2-cortisone) and 9,11,12,12 [^2^H]_4_-cortisol (d4-cortisol) were from Cambridge Isotope Laboratories. Solvents were high-performance liquid chromatography (HPLC) grade from Fisher Scientific.

### Clinical protocol

Screening tests were performed within the 2 weeks prior to the procedure. On the day of the study, subjects attended the Clinical Research Facility at around 0830 h. They were allowed to eat a light breakfast at home, but were only allowed water after their arrival at the research facility. They first underwent an ECG-gated phase contrast MR neck scan, which lasted 20–30 min. After this, a cannula was inserted into the left antecubital fossa and blood was taken to measure baseline endogenous steroids and their background isotopomers. At t = −5 min a 0.7 mg loading bolus of d4-cortisol was given intravenously (IV), followed by an infusion at 0.35 mg/h (15.94 nmol/min) at t = 0 min. A cannula was inserted into a vein in the dorsum of the right hand in a retrograde direction and when required the hand was placed in a hot box at 60°C to arterialize the blood. Arterialization of the blood was accepted if the oxygen saturation was >98%.

A jugular bulb cannula was inserted in the dominant internal jugular vein (assessed by MR venography) under ultrasound guidance. Placement was checked using a plain lateral C-spine x-ray: the tip of the catheter was visualized to ensure it was above the second cervical vertebra (16). The oxygen saturation of the blood was checked to ensure it was <85%.

At t = 145 min a 76.0 μg bolus of d2-cortisone was administered IV, followed by an infusion at 105.3 μg/h (4.88 nmol/min). From t = 180 min four sets of simultaneous blood samples were taken from the arterialized and jugular bulb cannulae at 10 min intervals (t = 180, 190, 200, and 210 min) into Lithium heparin and plasma separated and stored at −80°C until analysis.

### Magnetic resonance scanning

The MR imaging was performed with participants in the supine position on a 1.5 Tesla MR imaging unit (Signa HDxt, GE Healthcare) at the Brain Research Imaging Centre (www.bric.ed.ac.uk). An eight-channel neurovascular array coil was used. A noncontrast MR angiogram of the carotids from the level of the exterior auditory meatus down to the angle of the mandible was performed, followed by an MR venogram over the same area with a saturation band over the inferior aspect of the slices. The MR venogram was used to select the level for the ECG-gated phase-contrast MR scan by measuring a quarter of the way down from the jugular bulb to where the facial vein enters the internal jugular vein; axial images were taken perpendicular to the table. This level was chosen to ensure blood flow was measured in the vessel before any of the tributaries entered. The facial vein was the first, and to avoid measuring flow dynamics in the bulb itself, it may not be representative of the rest of the vessel, as there may be some pooling of blood where it first enters the bulb. Sixteen phase and magnitude images were taken at this level over one cardiac cycle. The flip angle was 25°, bandwidth 15.63 kHz, echo time (TE) 6 ms, repetition time (TR) 25 ms, and encoding velocity (V_enc_) 100 cm/s. Data were checked at the point of acquisition for any aliasing artifact. The field of view (FOV) was 25.6 cm × 25.6 cm and slice thickness of 5 mm. These images took approximately 8 min to acquire per patient, depending on the heart rate.

The image analysis software Medis Q flow (Medis medical imaging systems) was used to calculate jugular venous blood flow in mL/min on the side which was cannulated. The outline of the internal jugular vein was traced on each of the magnitude images from the cardiac cycle and blood flow calculated using data from the corresponding phase images. For completeness, the blood flow for the opposite internal jugular vein and both common carotid arteries were also measured.

### Laboratory analysis

Steroids (cortisol, d4-cortisol, 9,12,12 [^2^H]_3_-cortisol (d3-cortisol), cortisone and d2-cortisone) were quantified using liquid chromatography-tandem mass spectrometry (LC-MS). An internal standard solution [0.5 microg epicortisol (Steraloids), 0.25 microg 2,2,4,6,6,9,12,12 [^2^H]_8_-cortisone (d8-cortisone, Santa Cruz Biotechnology), and 0.25 microg 2,2,4,6,6,17A,21,21 [^2^H]_8_-corticosterone (d8-corticosterone, Cambridge Isotope Laboratories) with 9 μL methanol] was added to 1.5 mL of plasma, before 15 mL of chloroform was added for extraction of the steroids. The organic phase was then reduced to dryness under oxygen free nitrogen at 60°C before being reconstituted in the mobile phase [acetonitrile: water (35:65) with 0.1% formic acid]. Samples were injected on to a Sunfire C18 column (150 mm × 4.6 mm × 5 μm), with a column temperature of 10°C and a mobile phase flow rate of 1.5 mL/min using an Acquity Ultra Performance Liquid Chromatograph (Waters) coupled to a Qtrap 5500 mass spectrometer (AB Sciex). Ionization was achieved in the positive electrospray mode. The following transitions (precursor→product mass-to-charge ratios) used were as follows: cortisol (363→121), d2-cortisol (365→121), d3-cortisol (366→121), d4-cortisol (367→121), cortisone (361→77), d3-cortisone (364→164), and d2-cortisone (363→165). Steroid concentrations and tracer/tracee ratios were calculated from calibration curves and corrected for background isotopomer enrichments as described previously (17).

### Data analysis and kinetic calculations

Whole-body rate of appearance (Ra) of cortisol, d3-cortisol, and cortisone were calculated by dividing the rate of tracer infusion by the relevant tracer/tracee ratio (d4-cortisol/cortisol, d4-cortisol/d3-cortisol and d2-cortisone/cortisone, respectively) (3, 4). Brain tissue production of cortisol was calculated using data from arterial (A) and internal jugular vein (V) samples and corrected for cerebral blood flow using Eq. (1). Brain tissue production of d3-cortisol and cortisone were also calculated with Eq. (1), substituting arterial concentrations of d3-cortisol or cortisone and the relevant arterial and venous tracer:tracee ratios, as appropriate (3). Net release or uptake of cortisol across the brain was calculated using Eq. (2). Net release or uptake of d4-cortisol or cortisone were also calculated with Eq. (2), substituting arterial and venous concentrations of d4-cortisol and cortisone, respectively.

(1) $$\text{Tissue}\;\text{cortisol}\;\text{production}\;=\;\left(\left(\text{Blood}\;\text{flow}\times \left[{\text{cortisol}}_{\text{A}}\right]\right)\times \left(\frac{\text{d}4-\text{coristol}:\;\;{\text{coristol}}_{\text{A}}}{\text{d}4-\text{coristol}:\;\;{\text{coristol}}_{\text{V}}}\right)\right)-\left(\text{Blood}\;\text{flow}\times \left[{\text{cortisol}}_{\text{A}}\right]\right)$$

(2) $$\text{Net}\;\text{cortisol}\;\text{release}\;=\;\left(\left[{\text{cortisol}}_{\text{V}}\right]-\left[{\text{cortisol}}_{\text{A}}\right]\right)\;\times \;\left(\text{Blood}\;\text{flow}\right)$$

### Statistical analysis

There were no prior data for cortisol or cortisone production or uptake across the brain on which to base a power calculation, therefore we used previous data from a study on cortisol and cortisone production in skeletal muscle in healthy men (3). We calculated that in order to detect the same magnitude of difference from zero for net uptake or release across the brain of cortisol, d3-cortisol or cortisone, with a similar variance to that in skeletal muscle, would require sample sizes of 3, 5, and 7, respectively (for alpha 0.05, power 80% in a two-tailed test). We therefore considered a sample size of 8 to be reasonable.

Using SPSS, the student *t-*test was used to detect a difference from zero in brain production of cortisol, d3-cortisol, and cortisone. The small n in the study required the use of parametric tests, despite being unable to fully check the assumptions (18). However, on visual inspection there were no obvious outliers and the data appeared to meet the assumptions of normality.

## Results

Eight healthy men were recruited with a mean age of 38.1 years (sd 16.5) and mean BMI of 24.9 kg/m^2^ (sd 3.7). In 7 of the 8 subjects, the right internal jugular was found to be dominant or co-dominant on the MR scan and was used for cannulation and measurement of blood flow. In one subject, the left internal jugular vein (IJV) was dominant and was cannulated instead. Mean blood flow in the selected left internal jugular vein for all subjects was 481 mL/min (sd 232).

**Figure 2.**
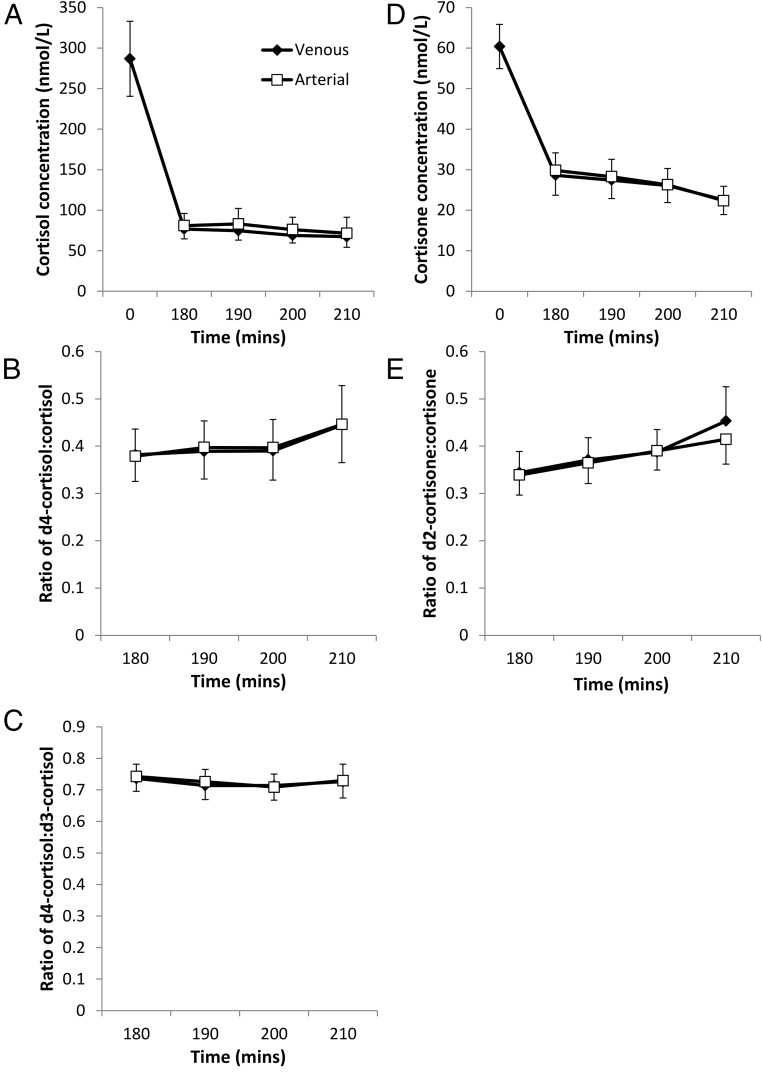
Arterialized and jugular venous steroid concentrations and tracer/tracee ratios during steady state stable isotope tracer infusion. Data are mean ± SEM for n = 8. (A) Cortisol concentration; (B) cortisone concentration; (C) d4-cortisol/cortisol ratio; (D) d4-cortisol/d3-cortisol ratio; (E) d2-cortisone/cortisone ratio. Statistical comparisons were made for the kinetic parameters derived from these “raw” data (Table 1).

Endogenous cortisol and cortisone concentrations in both arterialized and jugular venous samples reduced from baseline to 180 min of infusion, when the first set of arteriovenous samples were obtained, which is consistent with diurnal variation (Figure 2, A and D). In both arterialized and jugular venous samples, concentrations of cortisol and cortisone and tracer/tracee ratios were similar at all four sampling time points between 180 and 210 min of infusion, consistent with the steady state being achieved (Figure 2).

Mean values in arterialized samples in the steady state were used to calculate whole body rates of appearance of cortisol, d3-cortisol, and cortisone (Table 1), all of which were readily detectable, confirming technical success of the tracer infusions. Mean steady state data from arterialized and jugular venous samples were combined with blood flow measurements to calculate net release (or uptake) of cortisol and cortisone [Eq. (2)] and to estimate cortisol, d3-cortisol, and cortisone production across the brain [Eq. (1)] (Table 1). Surprisingly, d4-cortisol concentrations were higher in the jugular vein than arterialized samples, so that there was a net release of d4-cortisol across the brain in the steady state. No other indices of brain steroid release/uptake or production were significantly different from zero.

**Table 1.** Calculated Steady State Kinetics for Cortisol and Cortisone for the Whole Body and Brain

	Whole Body	Brain
Cortisol		
Rate of appearance of cortisol	47.5 (29.9–65.1)^a^	0.43 (−0.27–1.12)
Rate of appearance of d3-cortisol	22.5 (19.8–25.3)^a^	0.21 (−0.20–0.62)
Net brain release of cortisol		−0.54 (−3.75–2.66)
Net brain release of d4-cortisol		0.46 (0.22–0.70)^b^
Net brain release of d3-cortisol		0.12 (−0.36–0.60)
Cortisone		
Net rate of appearance of cortisone	14.4 (10.8–17.9)^a^	−0.02 (−0.35–0.30)
Net brain release of cortisone		−0.23 (−0.69–0.23)
Net brain release of d2-cortisone		−0.01 (−0.05–0.02)

Data are nmol/min shown as the mean (95% CI) for n = 8, except for net brain release of d2-cortisone, where reliable data could only be gained for n = 5. All other associations *P* > .05.

a *P* < .001.

b *P* < .005 vs 0 using the Student one-sample *t-*test.

## Discussion

We found no detectable interconversion of cortisol and cortisone across the human brain in eight healthy male volunteers, using deuterated glucocorticoid tracers and arteriovenous sampling. Previous studies using this approach have detected interconversion of cortisol plus or minus cortisone within liver, adipose tissue, and skeletal muscle (3, 5–7). Systemic cortisol/cortisone turnover values were similar in the subjects reported here to those observed in previous studies, so the approach was technically successful. The lack of statistically significant net production or release of cortisol, d3-cortisol or cortisone across the brain could not be attributed to variability in blood flow, either methodological due to the magnetic resonance method used, or biological due to asymmetry of internal jugular blood flow. Since we did not observe expected gradients in relevant steroid concentrations from arterialized to jugular venous blood, we sampled from the dominant jugular vein, and therefore from venous drainage of a substantial proportion of brain tissue. Any contribution of 11βHSD in the brain to whole body turnover between cortisol and cortisone is, therefore, negligible. It remains possible that, had we sampled from discreet brain subregions, 11βHSD activity might have been measurable, but this remains a speculation. The consequences of brain 11βHSD1 activity are likely to be confined to the subregions in which the enzyme is highly expressed (11).

In the absence of arteriovenous gradients in cortisol or cortisone concentrations, we did find a net release of d4-cortisol across the brain. It is unclear why there was release of tracer from the brain but it may indicate overpriming and higher circulating d4-cortisol levels earlier in the infusion, with resulting rerelease from the brain during steady state. However, this speculation cannot be tested in the absence of earlier samples.

One previous study has evaluated in vivo 11βHSD1 activity in the brain, by measuring peripheral venous plasma and cerebrospinal fluid (CSF) steroid concentrations during d4-cortisol infusion (19). Unfortunately, data were presented for only two subjects without administration of a potent 11βHSD1 inhibitor. Strangely, these subjects did not appear to reach steady state of tracer enrichment after 4 h of infusion and tracer/tracee ratios in plasma were not adjusted for d4-cortisol infusion to calculate the rate of appearance of cortisol and d3-cortisol and establish if the results were comparable with other published studies. CSF d3-cortisol concentrations were shown to be higher, relative to d4-cortisol, than plasma d3-cortisol concentrations, but only by comparing CSF data with plasma obtained 60 min earlier. This adjustment was applied on the grounds of closer correlation between plasma and CSF steroid concentrations separated by 60 min than those separated by shorter or longer intervals, but again indicates that steady state was not achieved in CSF. The authors attributed the apparent excess of d3-cortisol in CSF to brain 11βHSD1, partly on the basis that it was abolished in a dose-dependent fashion by administration of the 11βHSD1 inhibitor ABT384. However, ABT384 dramatically lowered plasma d3-cortisol concentrations, consistent with potent inhibition of systemic 11βHSD1 activity. The associated fall in CSF d3-cortisol concentrations, which fell below the limit of quantification in most samples, can be explained by the dramatic fall in plasma d3-cortisol without invoking any contribution of brain 11βHSD1. Against this background, it has not been established whether activity of 11βHSD1 in the brain is sufficient in magnitude to affect CSF cortisol concentrations.

We took a different approach to measuring steroids in jugular venous blood and calculating arteriovenous differences during steady state tracer infusion. With the measurement of blood flow, this allows absolute quantification of 11βHSD activities for all tissue draining to the cannulated internal jugular vein. 11βHSD1 is known to be expressed in specific neuronal subregions of the human brain: hippocampus (in particular the dentate gyrus and the cornu ammonis), prefrontal cortex, and the area of highest expression, the granule cell layer of the cerebellum (11). The venous drainage of the human brain is complex and appears to display considerable interindividual differences. Cerebellar venous blood drains to the superior and inferior cerebellar veins and usually ultimately into the internal jugular veins. However, posture affects cerebrovenous drainage such that in the supine position the internal jugular veins drain around 95% of blood flow from the intracerebral structures, while in the erect position this can drop to as little as 25%, with the remainder draining through the vertebral venous plexus (20). Our subjects were reclining at an angle of 45° for the arteriovenous sampling, but the measure of internal jugular vein blood flow was performed supine within a magnetic resonance imaging (MRI) machine. It could be that during blood sampling more of the cerebellar venous drainage was to the vertebral venous system rather than to the internal jugular veins, and that the brain 11βHSD1 was underestimated as a result. Moreover, the forebrain subregions where 11βHSD1 is expressed represent a minority, by mass, of forebrain tissue and hence any contribution of these subregions to the plasma steroid pool may be diluted by blood from elsewhere. More selective venous cannulation might therefore detect 11βHSD1 activity, which was not measurable here, but this is unlikely to be feasible in healthy volunteers.

As there were no previous data on brain release or uptake of cortisol or cortisone, sample size was calculated using data from a previous study on skeletal muscle (3). With the current novel results from the brain in hand, we have performed new power calculations which show that to demonstrate that the observed mean differences are statistically significantly different from zero for Ra cortisol, Ra d3-cortisol, and net Ra cortisone, we would need sample sizes of 40, 57, and 4006, respectively (for alpha 0.05, power 90% in a two-tailed test). We do not consider it justified to undertake invasive studies in this large number of subjects in order to more precisely quantify any small amount of cortisol production in the brain, having shown its mean magnitude to be negligible relative to other tissues.

If 11βHSD activity in the brain had been detectable with the approach used here, this could have provided a useful pharmacodynamic tool to quantify brain enzyme inhibition by selective 11βHSD1 inhibitors in development for treatment of dementia (14, 19). As things stand, neither this approach nor the CSF approach attempted by Katz et al (19) appear well-suited for this purpose in healthy volunteers. Given that 11βHSD1 expression increases with age in mice and is predictive of cognitive decline (12), it remains possible that arteriovenous sampling could be used to detect cortisol regeneration by 11βHSD1 in the brains of patients with dementia or with risk factors for cognitive decline (eg, diabetes, older age).
